# Oxidative stress-mediated HMGB1 biology

**DOI:** 10.3389/fphys.2015.00093

**Published:** 2015-04-07

**Authors:** Yan Yu, Daolin Tang, Rui Kang

**Affiliations:** Department of Surgery, University of Pittsburgh Cancer Institute, University of PittsburghPittsburgh, PA, USA

**Keywords:** HMGB1, ROS, inflammation, necrosis, apoptosis, autophagy, pyroptosis, NETosis

## Abstract

High mobility group box 1 (HMGB1) is a widely-expressed and highly-abundant protein that acts as an extracellular signal upon active secretion by immune cells or passive release by dead, dying, and injured cells. Both intracellular and extracellular HMGB1 play pivotal roles in regulation of the cellular response to stress. Targeting the translocation, release, and activity of HMGB1 can limit inflammation and reduce tissue damage during infection and sterile inflammation. Although the mechanisms contributing to HMGB1 biology are still under investigation, it appears that oxidative stress is a central regulator of HMGB1's translocation, release, and activity in inflammation and cell death (e.g., necrosis, apoptosis, autophagic cell death, pyroptosis, and NETosis). Thus, targeting HMGB1 with antioxidant compounds may be an attractive therapeutic strategy for inflammation-associated diseases such as sepsis, ischemia and reperfusion injury, arthritis, diabetes, and cancer.

## Introduction

High mobility group box 1 (HMGB1), a widely-expressed and highly-abundant protein, plays multiple roles in physiological and pathological processes and is implicated in human health and diseases (Kang et al., 2014a), especially aging (Huang et al., 2014b) and inflammation-associated diseases (Andersson and Tracey, 2011; Harris et al., 2012). HMGB1 was first identified in 1973 as a non-histone nuclear protein containing two DNA-binding HMG box domains (N-terminal A and central B) and an acidic C-terminal tail (Goodwin et al., 1973). Nuclear HMGB1, as an architectural factor, sustains chromosome structure and stability. Loss of HMGB1 *in vitro* and/or *in vivo* leads to a number of abnormalities in nuclear structure and function, such as genomic instability (Giavara et al., 2005), abnormal gene transcription (Rowell et al., 2012), impaired DNA damage response (Lange et al., 2008), impaired genome chromatinization (Celona et al., 2011), and inflammatory nucleosome release (Kang et al., 2014c). Under stress conditions, HMGB1 translocates from the nucleus to the cytosol and then releases into the extracellular space (Zhang et al., 2013). Cytosolic HMGB1, as a Beclin-1-binding protein, promotes autophagy, an evolutionarily-conserved and strictly-regulated lysosomal degradation pathway (Tang et al., 2010b). In some cases, HMGB1 presents in the cell membrane, contributing to neurite outgrowth, platelet activation, and cell adhesion. HMGB1 has not only intracellular functions, but also many extracellular functions such as cytokine and chemokine activity, which are mediated by HMGB1 receptors [e.g., receptor for advanced glycation end products (RAGE) (Hori et al., 1995), toll-like receptors (TLRs, such as TLR2, TLR4, and TLR9) (Park et al., 2004; Tian et al., 2007)] or endocytic HMGB1 uptake (Kang et al., 2014b; Xu et al., 2014a) to activate the downstream signaling pathway (e.g., nuclear factor-κB, interferon regulatory factor-3 and phosphatidylinositol 3-kinase) (Lotze and Tracey, 2005). Other aspects of HMGB1, such as post-translational modification [e.g., acetylation (Bonaldi et al., 2003) and phosphorylation (Youn and Shin, 2006), cleavage (Leblanc et al., 2014), and degradation (Li et al., 2011), as well as its binding partners (Bianchi, 2009), are also involved in the regulation of extracellular HMGB1 activity. HMGB1 global knockout mice die shortly after birth (Calogero et al., 1999), whereas HMGB1 conditional knockout/knockin mice exhibit a tissue-dependent phenotype under various stressors (Kitahara et al., 2008; Yanai et al., 2013; Huang et al., 2014a; Huebener et al., 2014; Kang et al., 2014c). Collectively, these findings suggest that HMGB1 plays a location- and modification-dependent role with fine-tuned mechanisms (Andersson et al., 2014).

Although the mechanisms for regulating HMGB1 release and activity vary depending on context, experimental studies reveal that oxidative stress is likely a common mechanism regulating HMGB1 translocation, release, and activity (Tang et al., 2011c). Oxidative stress is defined as an imbalance between the production of reactive oxygen species (ROS) and antioxidant defenses. Mild oxidative stress has been demonstrated to promote cell survival, whereas severe oxidative stress has been demonstrated to cause oxidative injury and even death. ROS, including superoxide anion (O^−^_2_), hydroxyl radical (OH), hydrogen peroxide (H_2_O_2_), and singlet oxygen (^1^O_2_), are primarily generated by mitochondria and NADPH oxidases (NOXs). Mitochondrion is not only an ROS production source, but also contains antioxidants. Mitochondrial dysfunction and NOX abnormality have been implicated in the etiology of numerous common diseases and aging (Finkel and Holbrook, 2000). This review describes recent advances in our understanding of oxidative stress-mediated regulation of HMGB1 biology in inflammation and cell death (Green et al., 2009; Linkermann et al., 2014).

## Oxidative stress-mediated HMGB1 release

### Infection

Sepsis is a systemic inflammatory response syndrome often caused by microbial infection or an inflammatory insult that induces the release of early and late response pro-inflammatory cytokines from immune (e.g., macrophages and monocytes) and non-immune (e.g., endothelial) cells (Angus and Van Der Poll, 2013). Early response cytokines [e.g., tumor necrosis factor and interleukin (IL)-1β] peak within the first hours after infection; circulatory levels then revert to near baseline in 3–4 h (Tracey and Cerami, 1993). HMGB1 is one of the delayed response cytokines, secreted by immune cells 20 h after activation with lipopolysaccharide (Wang et al., 1999). *In vivo*, HMGB1 is first detectable in the circulation 8 h after the onset of lethal endotoxemia and sepsis, subsequently increasing to plateau levels from 16 to 32 h (Wang et al., 1999). Targeting HMGB1 rather than early response cytokines provides a wider time window for clinical intervention to prevent lethal sepsis (Wang et al., 2008, 2014).

HMGB1 cannot be actively secreted via the classical endoplasmic reticulum-Golgi secretory pathway due to lack of a leader signal sequence. Instead, several mechanisms have been reported to be involved in active HMGB1 secretion in activated immune cells. These mechanisms include chromosome region maintenance 1 (CRM1)-mediated nuclear export (Bonaldi et al., 2003), the lysosome-mediated secretory pathway (Gardella et al., 2002), pyruvate kinase M2 isoform-mediated metabolism reprogramming (Yang et al., 2014), and double-stranded RNA-activated protein kinase (PKR)-mediated inflammasome activation (Lu et al., 2012), as well as oxidative stress-mediated mitogen-activated protein kinases and nuclear factor-κB pathways (Tang et al., 2007b). Antioxidants such as quercetin (Tang et al., 2009), edaravone (Kato et al., 2009), epigallocatechin gallate (Li et al., 2007), and resveratrol (Xu et al., 2014b) significantly inhibit HMGB1 release in animal models of sepsis, suggesting that oxidative stress mediates HMGB1 secretion during infection. Indeed, H_2_O_2_ at nontoxic doses (e.g., 0.0125–0.125 mM) can directly induce HMGB1 cytoplasmic translocation and activate release in macrophages and monocytes (Tang et al., 2007b). However, the effects of other ROS resources on HMGB1 release remain unexplained.

**Figure 1:**
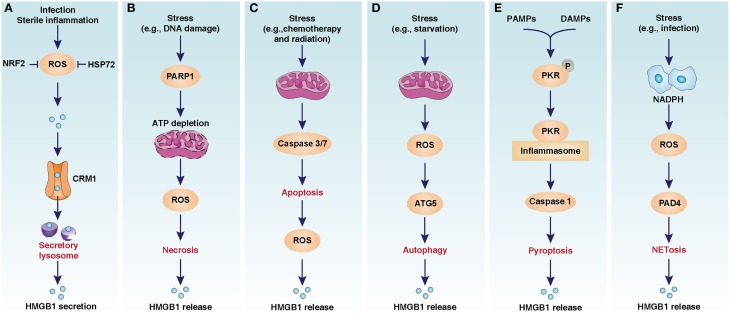
**Oxidative stress-mediated HMGB1 release in infection, inflammation, and cell death**. **(A)** CRM1-mediated nuclear export of HMGB1 in activated immune cells. **(B)** PARP1-medaited HMGB1 release in necrosis. **(C)** Caspase3/7-medaited HMGB1 release in apoptosis. **(D)** ATG5-medaited HMGB1 release in autophagy. **(E)** PKR-mediated HMGB1 release in pyroptosis. **(F)** PAD4-mediated HMGB1 release in NETosis. The image is modified from Kang et al. (2013).

Nuclear factor (erythroid-derived 2)-like 2 (NRF2) was recently identified as a general regulator of the anti-oxidative stress response by inducing the expression of a number of antioxidant response element-dependent genes such as heme oxygenase-1 (HO-1), a rate-limiting enzyme in the catabolism of heme. Suppression of NRF2 or HO-1 significantly increases oxidative injury and HMGB1 release, whereas upregulation of HO-1 inhibits HMGB1 release in infection (Kawahara et al., 2009; Takamiya et al., 2009; Tsoyi et al., 2009) (Figure 1A). Induction of HO-1 by NRF2 requires activation of p38 mitogen-activated protein kinases, confirming crosstalk between inflammation and the antioxidative response (Park et al., 2013). Thus, activation of the NRF2 pathway may have a therapeutic effect in inflammatory disease.

Heat shock proteins (HSPs) are highly-conserved and present in cells under normal conditions, but are overexpressed under some specific stress conditions such as temperature jump and infection. Induction of the heat shock response can suppress lipopolysaccharide-induced HMGB1 release in macrophages (Tang et al., 2005). HSP72, the main stress-induced HSP, inhibits oxidative stress-induced HMGB1 release by direct protein-to-protein interaction in macrophages (Tang et al., 2007a) (Figure 1A). Although these findings identify an essential role for HSP72 in blocking inflammation and preventing HMGB1 release in activated macrophages, it is not known whether an impaired heat shock response contributes to HMGB1 release in other cells.

### Sterile inflammation

Inflammation after tissue injury (e.g., ischemic, trauma, and transplant) in the absence of pathogens has been recognized for a long time and involves many changes, including release of endogenous damage-associated molecular patterns (DAMPs) (Tsung et al., 2014). Compared with pathogen-associated molecular patterns (PAMPs), which are non-self-molecules, DAMPs are self-molecules with the ability to activate inflammation via pattern recognition receptors. HMGB1 is a typical DAMP and mediates the sterile inflammatory response in response to ischemia/reperfusion (I/R) injury to multiple tissues such as liver (Tsung et al., 2005), heart (Andrassy et al., 2008), kidney (Wu et al., 2010), brain (Kim et al., 2006), and intestine (He et al., 2012). I/R injury is tissue damage caused when blood supply returns to tissue after a period of ischemia or lack of oxygen. Unlike in sepsis, HMGB1 levels are increased as early as 1 h after I/R injury and then increase in a time-dependent manner for up to 24 h (Tsung et al., 2005). Although several receptors (e.g., TLRs and RAGE) contribute to HMGB1 activity in I/R injury, TLR4 appears to be the dominant receptor for HMGB1-induced sterile inflammation in the liver (Tsung et al., 2005).

The release of HMGB1 during liver I/R is not only mainly caused by cell death, but may also be an active process regulated by ROS signaling (Tsung et al., 2007). Antioxidants N-acetyl-L-cysteine or Trolox inhibit hypoxic (1% oxygen)- or H_2_O_2_-induced HMGB1 release in hepatocytes *in vitro* and *in vivo* (Tsung et al., 2007). Remarkably, the calcium/calmodulin-dependent protein kinase type IV- and β-mediated calcium-dependent signaling pathways are downstream of oxidative stress, allowing HMGB1 release in I/R. Genetic and pharmacologic inhibition of calcium/calmodulin-dependent protein kinases inhibits HMGB1 release and protects against liver I/R injury (Tsung et al., 2007). Inhibition of HMGB1 activity by injection of neutralizing antibody partially abolishes the increase in liver manganese superoxide dismutase, a key mitochondrial antioxidant enzyme, after I/R (Pardo et al., 2008). In addition to inhibiting HMGB1 release in sepsis, activation of the NRF2-HO1 pathway also limits HMGB1 release and protects against I/R injury and other sterile inflammatory injuries (Yun et al., 2010; Wang et al., 2013) (Figure 1A). Considering these findings together, oxidative stress-induced calcium signaling facilitates HMGB1 release in sterile inflammation.

### Necrosis

In its default form, necrosis is a type of unexpected and accidental cell damage lacking the morphological characteristics of apoptosis or autophagy (Galluzzi et al., 2012). A very large number of chemicals or physical stimuli (e.g., toxins, radiation, heat, trauma, alcohol, drugs, and I/R injury) can cause necrosis. The release of intracellular contents, including HMGB1, after cellular membrane damage is the cause of inflammation in necrosis (Scaffidi et al., 2002). It is assumed that adenosine triphosphate (ATP) depletion from overactivation of ADP ribose polymerase (PARP) causes necrosis (Ha and Snyder, 1999). The best known member of the PARP family is a DNA nick sensor enzyme that becomes activated by DNA breaks. Excessive DNA damage during oxidative injury triggers PARP-mediated necrosis. Knockout of PARP1 in cells inhibits oxidative injury-induced HMGB1 release following necrosis in response to DNA damage (Ditsworth et al., 2007) (Figure 1B). PARP1 deficiency in mice and PARP inhibitors protect against stroke, I/R injury, sepsis, diabetes, and pancreatitis, suggesting that PARP1 is a potential target in inflammatory disease (Giansanti et al., 2010; Luo and Kraus, 2012).

In addition to the classic, unregulated form of necrosis, a form of regulated cell death, namely necroptosis, also exists (Galluzzi et al., 2012). The core components of the necroptosis machinery include receptor-interacting protein kinase (RIPK)-1, RIPK3, and the mixed lineage kinase domain-like protein (MLKL) (Linkermann and Green, 2014). Upon ligand binding, RIPK1 is recruited to tumor necrosis factor receptor superfamily and TLR complexes, promoting pro-survival and inflammatory signaling. RIPK1 also directly regulates caspase-8-mediated apoptosis or, if caspase-8 activity is blocked, RIPK3-MLKL-dependent necroptosis. Loss of RIPK1, RIPK3, and MLKL in mice protects against acute inflammation injury associated with decreased serum HMGB1 levels (Kaczmarek et al., 2013; Lau et al., 2013; Dannappel et al., 2014; Murakami et al., 2014). Oxidative stress is involved in execution of necroptosis downstream of RIPK1 activation (Vanden Berghe et al., 2014). Necrostatin 1, an inhibitor of necroptosis, inhibits ROS accumulation and HMGB1 release/activity in experimental animal models of sepsis and I/R injury (Duprez et al., 2011; Zhang et al., 2014). Moreover, the activity of PARP1 is regulated by RIPK1/RIPK3 in necroptosis, suggesting that interplay between PARP and RIPK can control necroptosis (Jouan-Lanhouet et al., 2012). One of the most active pursuits of cell death research is to elucidate the necroptotic pathway in the regulation of DAMP release, as well as the inflammatory response.

### Apoptosis

Apoptosis is the process of programmed cell death that may occur in a wide variety of physiological and pathological situations. It is often stated that apoptotic cell death doesn't provoke inflammation due to the cell membrane remaining intact and because apoptotic cells are rapidly phagocytosed by macrophages. Interestingly, HMGB1 was originally identified as a marker to distinguish between necrosis and apoptosis because HMGB1 is only released in necrosis, but not apoptosis, even after undergoing secondary necrosis and partial autolysis (Scaffidi et al., 2002). However, recent studies suggest that HMGB1 can be released in apoptosis by macrophages, cancer cells, and other cells (Bell et al., 2006; Jiang et al., 2007). DNA fragmentation is a marker of apoptosis that allows the release of nucleosomes (histones and DNA) and HMGB1 into the cytoplasm and extracellular space. Moreover, HMGB1 release is observed in macrophages during phagocytic clearance of apoptotic cells in sepsis (Qin et al., 2006). HMGB1 released from apoptotic cell death is an important mediator of immunogenic cell death in cancer therapy by TLR4 (Apetoh et al., 2007).

The activity of HMGB1 release in apoptosis is context-dependent. Oxidative stress is likely to diminish the immune activity of HMGB1 in apoptosis. Caspases, a family of cysteine proteases, are the central regulators of apoptosis. Activated caspase-3 and -7 in the mitochondrial apoptotic pathway can cleave NADH dehydrogenase Fe-S protein-1, the 75 kDa subunit of respiratory complex I. This process leads to a loss of ATP and the generation of ROS. As a redox-sensitive protein, HMGB1 contains three cysteines: C23, C45, and C106. The oxidation of Cys106 in apoptosis abolishes the ability of HMGB1 to activate dendritic cells, the specialized antigen-presenting cells that initiate and direct T-cell immunity (Kazama et al., 2008). In contrast, mutation of a single amino acid in the caspase cleavage site of NADH dehydrogenase Fe-S protein-1 can inhibit ROS production and restore the immune activity of released HMGB1 during apoptosis (Kazama et al., 2008) (Figure 1C). Thus, the redox status of HMGB1 in apoptosis appears to determine whether cell death is tolerogenic or immunogenic.

### Autophagy

Autophagy is a conserved, intracellular, lysosome-dependent degradation process that allows the degradation and recycling of cellular components such as proteins and organelles. Autophagy-related genes (Atg) are essential regulators of autophagy in development and most other stages of adult life. Homozygous genetic knockouts of most Atg genes (e.g., Atg3, Atg5, Atg6, Atg7, Atg9, and Atg16L1) in mice are developmentally lethal (Mizushima and Levine, 2010). In addition to physiological function, dysfunction of autophagy is implicated in human diseases such as autoimmune diseases, inflammatory diseases, and cancer. Autophagy is upregulated in response to various stressors, including oxidative stress. In many cases, upregulated autophagy promotes cell survival during various stress conditions, while excessive or uncontrolled autophagy promotes cell death in some cases (Kroemer et al., 2010). The concept of autophagic cell death was first presented based on observations of increased morphological features (e.g., accumulation of autophagic vesicles) in dying cells (Galluzzi et al., 2012). Moreover, selective overexpression of autophagy can cause a special type of regulated cell death termed “autosis,” which is mediated by the Na^+^, K^+^-ATPase pump (Liu et al., 2013). This report has led to increasing concerns about the pathogenic role of autophagic cell death, although controversy still exists.

HMGB1 plays location-dependent roles in the induction of autophagy. Cytoplasmic HMGB1 promotes starvation- and oxidative stress-induced autophagy by binding Beclin-1 (Tang et al., 2010b), a critical regulator of autophagy, as well as apoptosis (Kang et al., 2011). Extracellular HMGB1 induces autophagy by binding its receptor RAGE (Tang et al., 2010a). Nuclear HMGB1 regulates autophagy by inducing expression of heat shock protein β-1, which allows membrane dynamic trafficking during autophagy and mitophagy (Tang et al., 2011a). Like other ATGs, it was recently suggested that HMGB1-independent autophagy exists in some tissues. The mechanisms of the HMGB1-dependent and -independent autophagic signaling pathways remain to be defined.

HMGB1 release is observed in macrophages, fibroblasts, and cancer cells during autophagy and autophagic cell death (Thorburn et al., 2009; Tang et al., 2010b; Dupont et al., 2011) (Figure 1D). Superoxide dismutases (SOD), including SOD1, SOD2, and SOD3, can efficiently catalyze the dismutation of superoxide anions. Antioxidant (e.g., N-acetyl-L-cysteine) SOD1 RNAi, and SOD2 RNAi limit the translocation and release of HMGB1 in starvation-induced autophagy, suggesting that oxidative stress is involved in starvation-mediated HMGB1 release (Tang et al., 2010b). Moreover, H_2_O_2_ and loss of SOD1-mediated oxidative stress promote cytosolic HMGB1 expression and extracellular release (Tang et al., 2011b). Inhibition of HMGB1 release or loss of HMGB1 decrease the number of autolysosomes and autophagic flux in human and mouse cell lines under conditions of oxidative stress (Tang et al., 2011b). Collectively, HMGB1 appears to serve as an important autophagic sensor in oxidative stress.

### Pyroptosis

Pyroptosis is a caspase-1 or caspase-11-dependent regulated type of cell death that plays a central role in inflammation and immunity. According to their function, caspases are divided into apoptotic (caspase-3, -6, -7, -8, and -9 in mammals) and inflammatory (caspase-1, -4, -5, -12 in humans and caspase-1, -11, and -12 in mice) families (McIlwain et al., 2013). Compared with caspase-11-dependent pyroptosis (Broz and Monack, 2013), caspase-1-dependent pyroptosis is well-characterized (Bergsbaken et al., 2009). Caspase-1 is activated during pyroptosis by inflammasome, a large supramolecular complex largely composed of dimers of the adaptor protein ASC (apoptosis-associated speck-like protein containing a caspase recruitment domain). Four subfamilies of inflammasome are defined; namely, NLRP1, NLRP3, NLRC4, and absent in melanoma 2 (AIM2). These inflammasomes detect PAMPs and DAMPs in infection and sterile inflammation (Schroder and Tschopp, 2010). Mitochondrial and NOX-mediated oxidative stress is an initial signal to induce inflammasome activation. In particular, the generation of ROS via NOX causes thioredoxin-interacting protein to associate with NLRP3, which facilitates inflammasome formation (Zhou et al., 2010). Finally, caspaspe-1 is activated by inflammasome and then promotes the procession and release of the highly pro-inflammatory cytokines IL-1β and IL-18, as well as HMGB1. Inhibition of inflammasome activation decreases serum HMGB1 level and protects against liver ischemic injury and pancreatitis (Kamo et al., 2013). Interestingly, HMGB1 can activate NLRP3 and AIM2 inflammasome after binding various DNA in a TLR9- or RAGE-dependent manner, suggesting a feedback loop between HMGB1 and inflammasome (Tian et al., 2007). Alternatively, HMGB1 endocytosis results in NLRP3 inflammasome-independent and lysosome-dependent caspase-1 activation (Xu et al., 2014a). Thus, HMGB1 acting through different signaling pathways sequentially induces pyroptosis.

Like all caspases, caspase-1 can recognize a unique cleavage site in substrate. A recent study identified that HMGB1 is a direct substrate of caspase-1, but not other caspases (-2, -3, -5, -7, -9, or -11) (Leblanc et al., 2014). In contrast to HMGB1 oxidization-mediated immune tolerance in apoptosis following caspase-3 and -7 activation (Kazama et al., 2008), HMGB1 cleavage by caspapse-1 generates an active A-box peptide and then induces inflammation by RAGE (Leblanc et al., 2014). These findings suggest that activation of different caspapses determines the immune activity of HMGB1 in death. In addition to the classical inflammasome components, PKR is a novel binding protein of inflammasome to regulate inflammation. Inhibition of PKR expression or activity inhibits inflammasome activation and HMGB1 release in macrophages (Lu et al., 2012) (Figure 1E). Mitophagy or autophagy generally inhibits inflammasome activation by inhibition of mitochondrial ROS production or direct degradation of the core components of inflammasome, respectively (Nakahira et al., 2011; Deretic et al., 2013). Correlations between autophagy, pyroptosis, and DAMP release have not yet been studied in human disease.

### NETosis

NETosis is a antimicrobial cell death that occurs primarily in polymorphonuclear leukocytes or neutrophils in response to infection (Remijsen et al., 2011). The discovery of neutrophil extracellular traps (NETs), DNA-protein structures released by neutrophils (Brinkmann et al., 2004), was originally studied. NETs can capture and kill invading microorganisms and pathogens without using the mechanism of phagocytosis. In addition to infection and PAMPs (e.g., lipopolysaccharide), several cytokines (e.g., IL-8 and tumor necrosis factor) and DAMPs (e.g., uric acid) can induce NET formation in neutrophils and immune cells (Brinkmann and Zychlinsky, 2012; Yipp and Kubes, 2013). Accumulating evidence indicates that NETs are released in the context of cell death, a process called NETosis (Steinberg and Grinstein, 2007). NETosis not only involves the innate host defense, but also promotes thrombosis and I/R injury (Martinod and Wagner, 2014; Savchenko et al., 2014). Importantly, tumor-induced neutrophils are more sensitive to NETosis than normal neutrophils, suggesting that NETosis is involved in cancer progression (Demers et al., 2012).

In addition to histones and DNA, HMGB1 is a nuclear component of NETs regulated by several different mechanisms, including autophagy (Mitroulis et al., 2011). Extracellular HMGB1 can also induce NET formation by autophagy (Tadie et al., 2013; Maugeri et al., 2014a) and exhibits bacterial killing ability (Zetterstrom et al., 2006). ROS production by activation of NOX contributes to NETosis (Figure 1F). Interestingly, extracellular HMGB1 may directly induce NOX activation in neutrophils and mediate hemorrhagic shock in a TLR4-dependent manner (Fan et al., 2007). Thus, release of HMGB1 in inflammation may amplify oxidative stress and NETosis. Although eptidylarginine deiminase 4-mediated histone citrullination is a critical step to regulate histone release in NETosis and other types of cell death, future work will address whether HMGB1 is a regulator of eptidylarginine deiminase 4, which is required for NET-mediated innate immunity and thrombosis.

## Redox modification of HMGB1 activity in inflammation and cell death

In inflammation, extracellular HMGB1 has the ability to stimulate cell migration and cytokine production in receptor-dependent and -independent manners. This HMGB1 activity is determined by its redox status of cysteines (C23, C45, and C106). Three redox forms of HMGB1 have been identified: all-cysteine-reduced HMGB1, disulfide HMGB1, and all-cysteine-oxidized HMGB1. All-cysteine-reduced HMGB1 forms a heterocomplex with C-X-C motif chemokine 12 and then binds to C-X-C chemokine receptor type 4 to induce cell migration and inflammatory cell recruitment (Venereau et al., 2012). Surprisingly, this all-cysteine-reduced HMGB1 can't induce cytokine production in immune cells. In contrast, reduced C106 is required for binding of HMGB1 to TLR4 to trigger cytokine release in macrophages (Yang et al., 2010). Subsequent studies demonstrated that a disulfide bond between C23 and C45 is also required for HMGB1's cytokine-inducing activity (Venereau et al., 2012; Yang et al., 2012). Finally, all-cysteine-oxidized HMGB1 does not show cytokine or chemotactic activity (Venereau et al., 2012). However, other studies have revealed that oxidized HMGB1 still has the ability to activate neutrophils and vascular endothelial cells and trigger age-related inflammation (Davalos et al., 2013; Maugeri et al., 2014b). The reasons for the discrepancies between studies are unclear, but may be the result of differences in purity of HMGB1 redox protein preparations and the used dose. More convincing evidence is needed to develop specific neutralizing antibody to recognize these three different redox HMGB1 proteins or use mice with HMGB1 cysteine genetic mutation. Overall, the question of whether different redox forms of HMGB1 are good or bad is inadequately framed.

In addition to inflammation, redox modifications are crucial for the function of HMGB1 in DNA repair and autophagy. Compared with oxidized HMGB1, reduced HMGB1 exhibits a stronger affinity for distorted DNA structures, but their functional roles are not yet clear (Park and Lippard, 2011). Replacement of Cys23 and/or 45 with serines did not affect the nuclear distribution of the mutant proteins, while C106S and triple cysteine mutations impaired nuclear localization of HMGB1 (Hoppe et al., 2006), allowing entry of some of the protein into the cytosol to induce autophagy (Tang et al., 2010b). A disulfide bond between C23 and C45 is required for binding of HMGB1 to Beclin-1 to induce autophagy (Tang et al., 2010b). Moreover, extracellularly-reduced HMGB1 promotes autophagy in a RAGE-dependent manner, whereas oxidized HMGB1 triggers caspase-dependent apoptosis in cancer cells (Tang et al., 2010a). Whether and how these redox modifications of HMGB1 can affect cell-to-cell communication in the tumor microenvironment remain to be elucidated (Kang et al., 2013).

## Conclusions

Since the discovery of HMGB1 in 1973 (Goodwin et al., 1973), the multi-faceted HMGB1 has recently been identified as an important mediator of infection and sterile inflammation, which has initiated a new field of translational medicine for targeting HMGB1 in disease (Kang et al., 2014a). What lies ahead? An improved understanding of the underlying regulation mechanisms of HMGB1 expression, translocation, and release; how HMGB1 contributes to the pathophysiology of human health and diseases; and the development of more specific and less toxic compounds will benefit many more patients and improve their quality of life. In particular, oxidative stress-associated human aging and many diseases have been increasingly recognized as central regulators of HMGB1 biology in infection, sterile inflammation, and cell death types such as necrosis, apoptosis, autophagic cell death, pyroptosis, and NETosis (Tang et al., 2011c). Different stressors may cause different stress responses, cell deaths, and immune responses. Activation of autophagy possibly mediates the crosstalk between cell death, inflammation, and DAMP release during stress (Levine et al., 2011; Deretic et al., 2013; Zhang et al., 2013; Green and Levine, 2014). Targeting HMGB1 via antioxidant compounds such as N-acetyl-cysteine protects against inflammation-associated diseases in animal experiments. However, these studies seem to contradict human clinical trials in which antioxidant therapy proved largely ineffective in treating various pathologies. One possible reason for this is that the reactivity of ROS is orders of magnitude too fast. This leaves a narrow therapeutic window for administration of antioxidant treatment, and antioxidant therapies are not effective when delivered after the acute cytokine response has occurred. Alternatively, reducing HMGB1 release and the inflammatory response through bolstering intracellular enzymatic antioxidants (e.g., HO-1, SODs, and catalase) or blocking their enzymatic production may be an attractive strategy. In addition to blocking HMGB1 release, HMGB1 neutralizing antibody and A box protein can block activity of extracellular HMGB1 in animal experiments. Further investigation is needed to evaluate these therapies and their possible roles in clinical practice. Although much attention has been paid to the different modifications of HMGB1 within the past 5 years, monitoring the intracellular and extracellular redox statuses of HMGB1 in disease still presents a major challenge (Antoine et al., 2014; Janko et al., 2014). Additionally, much remains unknown about how individual signal transduction pathways are regulated upon activation by different redox forms of HMGB1.

### Conflict of interest statement

The authors declare that the research was conducted in the absence of any commercial or financial relationships that could be construed as a potential conflict of interest.
